# Supplementary material for: Catalytic Oxidation of Acetone over MnOx-SiO2 Catalysts: An Effective Approach to Valorize Rice Husk Waste
Source: Materials (Basel). 2024 Dec 12;17(24):6069. doi: 10.3390/ma17246069 (PMC11676639; doi:10.3390/ma17246069)
Supplement: Supplementary file 1 [file materials-17-06069-s001.zip › materials-3344040-supplementary.pdf]

Supplementary Material

Catalytic Oxidation of Acetone over MnO_x-SiO_2 Catalysts: An Effective Approach to Valorize Rice Husk Waste

Mauricio Cardoso [1], Patrice Portugau [1], Carolina De Los Santos [1], Ricardo Faccio [2], Hilario Vidal [3], José Manuel Gatica [3], María del Pilar Yesté [3], Jorge Castiglioni [1] and Martin Torres [1,*]

1. Área Fisicoquímica, Facultad de Química, Universidad de la República, Gral. Flores 2124, Montevideo 11800.
2. Área Física, Facultad de Química, Universidad de la República, Gral. Flores 2124, Montevideo 11800, Uruguay.
3. Departamento C.M. I.M. y Química Inorgánica, Universidad de Cádiz, 11510 Puerto Real, Spain.

* mtobru@fq.edu.uy

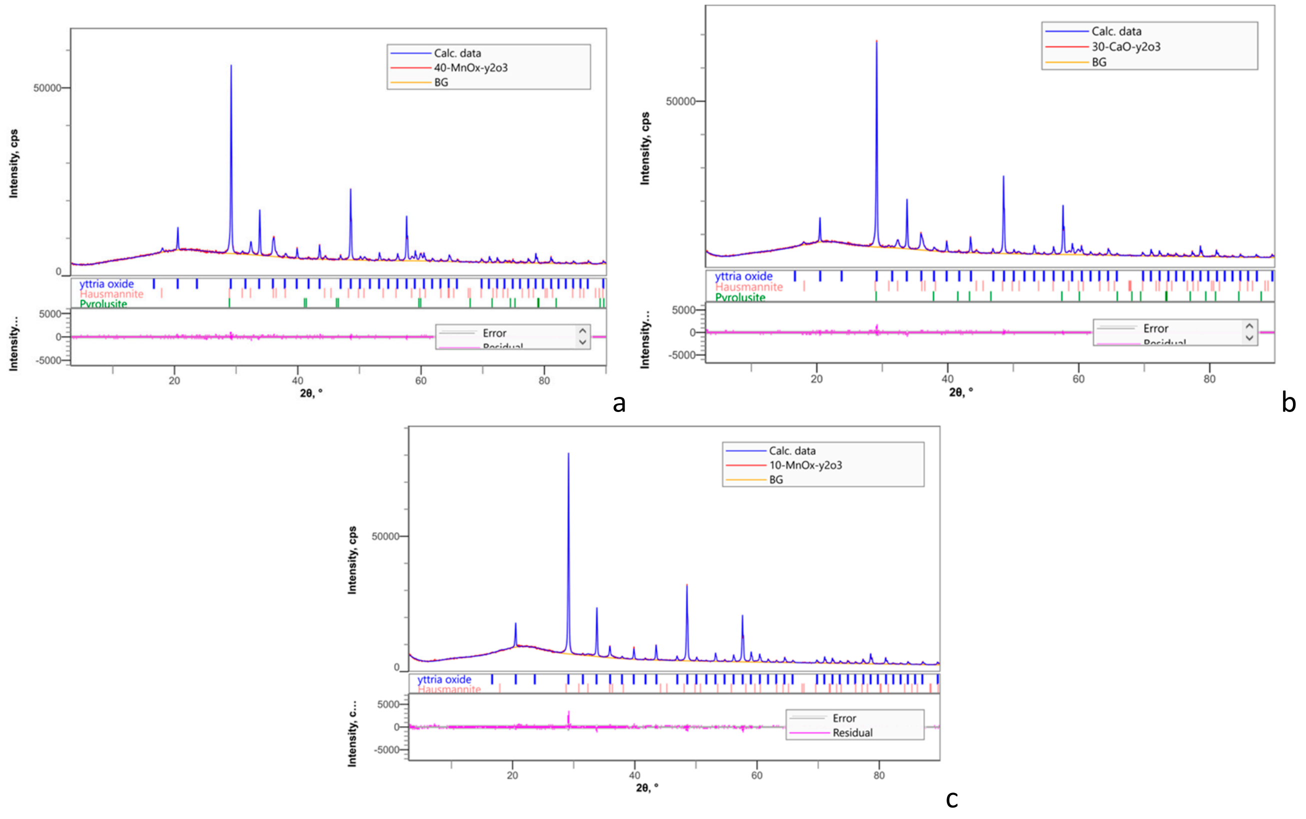

Figure S1. Rietveld profile fitting for a) Mn40RHS, b) Mn30RHS and c) Mn10RHS samples including yttria.

Table S1. Summary of main statistics of the Rietveld refinement for Mn40RHS, Mn30RHS and Mn10RHS.

	Rwp, %	Rp, %	S	χ^2
Mn10RHS	3.02	2.36	0.76	0.58
Mn30RHS	2.88	2.27	0.73	0.53
Mn40RHS	2.95	2.31	0.72	0.52
